# Nonlinear relationship between the ratio of remnant cholesterol to high-density lipoprotein cholesterol and hyperuricemia in the general population

**DOI:** 10.1097/MD.0000000000048360

**Published:** 2026-04-10

**Authors:** Yanyan Xuan, Jinbo Yu, Fangfang He, Yuanyuan Cen, Haibo Chen, Qi Yao

**Affiliations:** a Department of Hospital Infection, The First Affiliated Hospital of Ningbo University, Ningbo, Zhejiang Province, China; b Department of Hepatology, The First Affiliated Hospital of Ningbo University, Ningbo, Zhejiang Province, China; c Department of Geriatrics Medicine, The First Affiliated Hospital of Ningbo University, Ningbo, Zhejiang Province, China; d Department of Respiratory and Critical Care Medicine, Cixi Longshan Hospital, Ningbo, Zhejiang Province, China; e Department of Rheumatology and Immunology, The First Affiliated Hospital of Ningbo University, Ningbo, Zhejiang Province, China.

**Keywords:** high-density lipoprotein cholesterol, hyperuricemia, nonlinear, remnant cholesterol, uric acid

## Abstract

The global prevalence of hyperuricemia (HUA) is increasing year on year, raising urgent public health concerns. This study aimed to investigate the relationship between the remnant cholesterol to high-density lipoprotein cholesterol (RC/HDL-C) ratio and HUA. Data from 2795 participants in the 2017 to 2018 National Health and Nutrition Examination Survey were used for this cross-sectional study. Subgroup analyses were performed, stratified by age, sex, race, and body mass index (BMI), to evaluate the association between the RC/HDL-C ratio and the risk of HUA and serum uric acid levels, using adjusted multivariate logistic and linear regression analyses. The nonlinear relationship between the RC/HDL-C ratio and HUA risk was also tested using generalized additive models. Of the 2795 participants, 583 were diagnosed with HUA. A higher RC/HDL-C ratio is associated with an increased risk of HUA, model 1 (odds ratio [OR] = 3.677, 95% confidence interval [CI]: 2.841, 4.760), model 2 (OR = 3.609, 95% CI: 2.739, 4.755), and model 3 (OR = 2.551, 95% CI: 1.834, 3.548). Stratified analysis revealed that this association was particularly evident among women, individuals over 60 years of age, Other Hispanic individuals, and individuals with a BMI below 25 kg/m^2^. Furthermore, nonlinear correlation analysis revealed a threshold saturation effect between the RC/HDL-C ratio and HUA, with an inflection point at 0.400. In the American population, an increase in the RC/HDL-C ratio is independently related to an increased risk of HUA and higher serum uric acid levels. This correlation remains stable when further stratified by gender, age, and BMI.

## 1. Introduction

Hyperuricemia (HUA) is a metabolic syndrome (MetS) characterized by excessive production or insufficient excretion of serum uric acid (SUA) due to a disorder of purine nucleotide metabolism.^[1,2]^ HUA is the main factor leading to long-term systemic inflammation in patients with gout.^[3,4]^ An inflammatory reaction induced by uric acid (UA) salts in patients with asymptomatic HUA can lead to the development of metabolic-related diseases. As demonstrated in previous studies, SUA is associated with several medical conditions, including hyperlipidemia, MetS, hypertension, obesity, diabetes, metabolic fatty liver disease, cardiovascular disease, and other diseases. Furthermore, it has been observed to be closely related to the poor prognosis of cardiovascular and cerebrovascular diseases and renal diseases.^[5,6]^ Previous studies indicated that the global prevalence of HUA has risen steadily since the 1960s, reaching 21% worldwide in 2016.^[7,8]^ According to the National Health and Nutrition Examination Survey (NHANES) data, around 47.1 million American adults met the diagnostic criteria for HUA between 2015 and 2016, corresponding to a prevalence rate of 20.2% among males and 20.0% among females in the general population.^[9]^ However, as patients with HUA often exhibit no apparent symptoms in the early stages, the condition has not received enough attention. Furthermore, HUA is a complex condition influenced by multiple risk factors. Consequently, there is an urgent need for a simple, rapid screening method to promptly identify individuals at high risk of HUA, enabling targeted interventions to prevent related diseases.

In recent years, extensive research has focused on the role of lipid metabolism abnormalities in HUA and gout, while also investigating the predictive capacity of various lipid markers for HUA risk.^[10,11]^ Remnant cholesterol (RC) is a relatively new nontraditional lipid marker that was proposed in recent years. RC is a lipoprotein that promotes atherosclerosis and is mainly found in triglycerides (TG)-rich lipoproteins. In the fasting state, it comprises primarily very low-density lipoprotein cholesterol (LDL-C) and intermediate-density lipoprotein cholesterol, alongside chylomicron remnants in the non-fasting state.^[12]^ In 2019, the European Atherosclerosis Society’s guidelines for dyslipidemia management recommended calculating RC by subtracting high-density lipoprotein cholesterol (HDL-C) and LDL-C from total cholesterol (TC).^[13]^ Research indicates elevated RC levels are associated with low-grade inflammation and insulin resistance (IR).^[14–16]^ The incidence of hyperlipidemia, obesity, hypertension, cardiovascular disease, and type 2 diabetes mellitus (T2DM) rises as RC levels increase.^[11,17–19]^ Recent cohort and cross-sectional studies conducted across multiple countries and populations have confirmed the association between elevated RC levels and HUA. These studies have identified elevated RC levels as an independent risk factor for HUA.^[20–24]^

Dyslipidemia is a primary risk factor for HUA, characterized by reduced HDL-C levels, elevated TG, and LDL-C levels. Among these, HDL-C is recognized as a protective factor against HUA.^[25]^ Several previous studies have indicated a negative correlation between HDL-C and SUA levels, with HDL-C exerting a protective effect against HUA.^[10,11,26]^ Nevertheless, these studies only consider the relationships between individual lipid markers and HUA.^[27]^ However, lipid and lipoprotein ratios may reflect interactions between lipid components. Recent investigations have revealed that increased RC levels in the blood may lead to decreased HDL-C levels through plasma exchange of TG and cholesterol between HDL-C and RC.^[28]^ Consequently, combining RC and HDL-C measurements may be more effective than assessing lipid levels alone for predicting HUA risk. We therefore hypothesize that the ratio of RC to HDL-C is closely associated with HUA.

This study is based on data from the NHANES. It aims to evaluate the relationship between the remnant cholesterol to high-density lipoprotein cholesterol ratio (RC/HDL-C) ratio, SUA levels, and the risk of developing HUA in the general US population. Subgroup analyses were also conducted according to factors such as gender, age, ethnicity, and body mass index (BMI).

## 2. Methods

### 2.1. Study design and population

**Figure 1.**
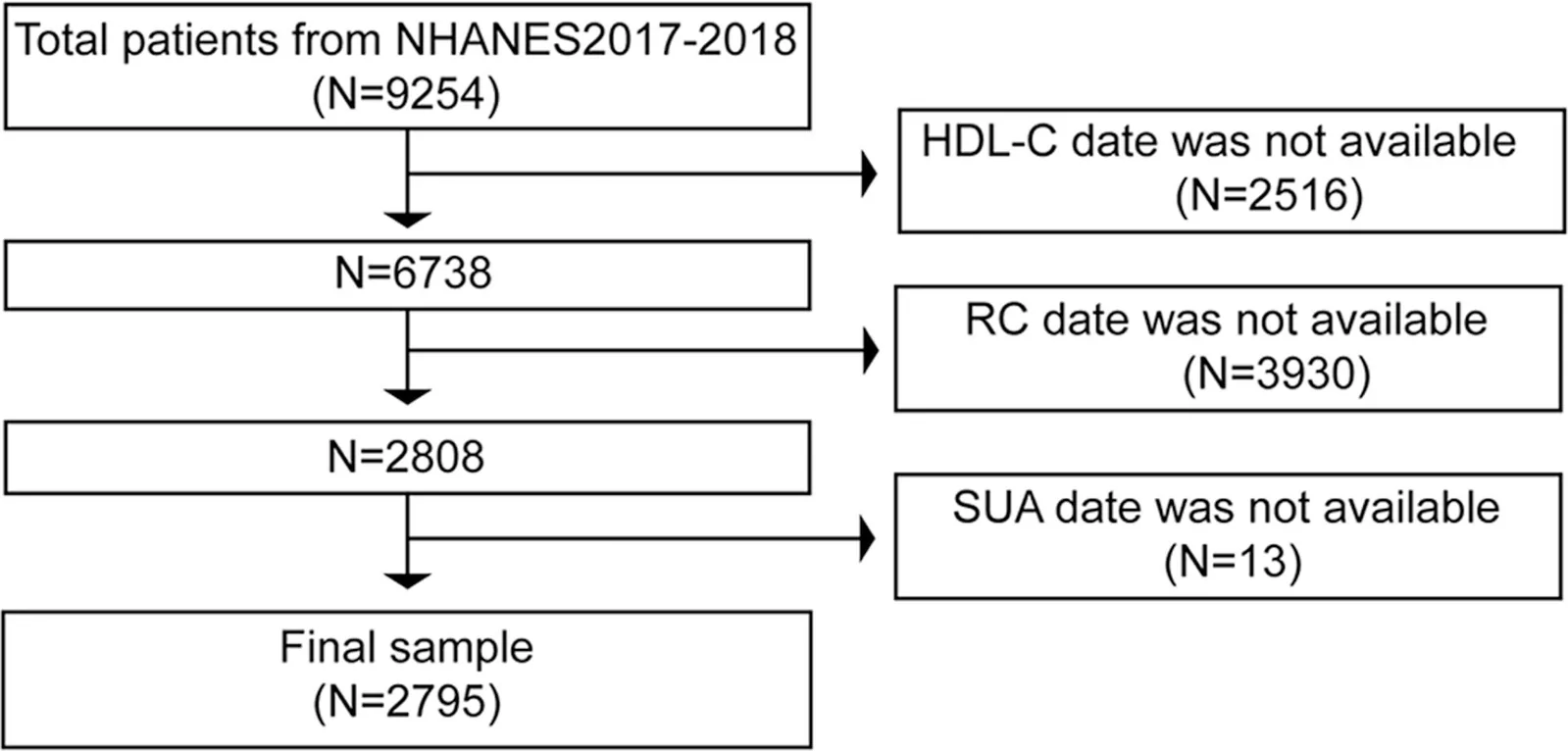
Flowchart of the study participants. HDL-C = high-density lipoprotein cholesterol, NHANES = National Health and Nutrition Examination Survey, RC = remnant cholesterol, SUA = serum uric acid.

The data originate from the 2017 to 2018 study of the NHANES, a cross-sectional, comprehensive survey conducted by the National Center for Health Statistics (NCHS). The NHANES employs a stratified, multistage probability sampling design, encompassing individuals representative of the general, noninstitutionalized population of the United States. This database has been widely used in epidemiology and health research. This is achieved through participant questionnaires, household interviews, physical examinations, specialized measurements, and laboratory tests. The entire NHANES study design and associated data are freely accessible online at https://www.cdc.gov/nchs/nhanes/. Researchers worldwide may utilize NHANES data. The NCHS Ethics Committee approved the methods and materials employed in the survey, and all information collected during the study is strictly confidential. Among these subjects, we eliminated 3353 participants with SUA deficiency. Next, we eliminated the 6 individuals for whom HDL-C values were unavailable and 3100 individuals for whom RC values were unavailable. Consequently, 2795 subjects were involved in the final analysis (Fig. 1).

### 2.2. Definition of RC and HUA

RC was calculated using the following formula: RC (mg/dL) = TC (mg/dL) − HDL-C (mg/dL) − LDL-C (mg/dL).^[13]^ The RC/HDL-C ratio was calculated by dividing RC by HDL-C. The UA level was measured using a Beckman Coulter UniCel DxC800 instrument (Beckman Coulter, Indianapolis). HUA was defined as an SUA level of ≥7.0 mg/dL in males and ≥6.0 mg/dL in females.^[9]^

### 2.3. Variables

RC/HDL-C ratio was considered the independent variable, and the level of SUA and risk of HUA were considered dependent variables. The confounders employed in this investigation were derived from established variables identified in prior research and clinical practice. Gender, race, the status of smoking, the status of drinking, physical activity, the status of hypertension, and the status of T2DM were the categorical factors used as covariates in our study. We also included the following continuous variables as covariates in our analysis: liver enzymes, serum lipids, high-sensitivity C-reactive protein (hs-CRP), fasting glucose, fasting insulin, glycosylated hemoglobin (HbA1c), estimated glomerular filtration rate (eGFR), blood urea nitrogen (BUN), systolic/diastolic blood pressure (SBP/DBP), and BMI were adjusted.

Race/ethnicity was determined using self-reported data from the NHANES demographic questionnaire. Participants were classified into 6 mutually exclusive groups: Mexican American; Other Hispanic, defined as individuals of Hispanic or Latino origin excluding Mexican Americans (primarily including Puerto Ricans, Cubans, Central Americans, and South Americans); non-Hispanic White; non-Hispanic Black; non-Hispanic Asian; and Other race, which includes American Indian/Alaska Native, Native Hawaiian/Other Pacific Islander, or Other single or multiple races. Smoking status was determined using data from the smoking questionnaire. Participants were categorized as never smokers (fewer than 100 cigarettes smoked in their lifetime), former smokers (100 or more cigarettes smoked in their lifetime but not currently smoking), or current smokers (100 or more cigarettes smoked in their lifetime and currently smoking every day or some days). Heavy alcohol consumption was defined as consuming more than 2 standard drinks per day for men or more than 1 standard drink per day for women, based on average alcohol intake over the past 12 months. If any of the following criteria were present, hypertension was defined as a SBP of more than 140 mm Hg or a DBP of more than 90 mm Hg was measured; currently, antihypertensive medications are being taken; or self-reported hypertension. The following criteria were used to diagnose T2DM: self-reported diabetes; antidiabetic medication use; a measurement of fasting plasma glucose of 7 mmol/L or higher; a random plasma glucose of ≥11.1 mmol/L; and a level of ≥6.5% for HbA1c. The BMI of the subject was calculated by dividing their weight in kilograms by the square of their height in meters. Furthermore, the exercise degree was evaluated and classified as active, moderate, or inactive, per the established physical exercise guidelines.^[29]^

### 2.4. Statistical analysis

The data were analyzed using the R language 4.2.0 (R Foundation for Statistical Computing, Vienna, Austria, http://www.Rproject.org). The data analysis employed the weighting recommended by the NCHS to ensure that the findings accurately represented the US population. Statistical significance was defined as a *P* value < .05. In the event of a normal distribution of the variables, the weighted mean ± standard deviation was utilized to express the data. In instances where continuous variables were not normally distributed, medians or interquartile ranges were reported. Categorical variables were represented as weighted proportions and frequencies. To examine the relationship between the RC/HDL-C ratio and HUA, 3 logistic regression models were developed after the RC/HDL-C ratio was classified quarterly. First, none of the covariates were adjusted. Second, adjustments were made for age, gender, and race. Third, adjustments were made for all relevant variables: age, gender, race, hypertension, physical activity, BMI, T2DM, drinking status, smoking status, DBP, SBP, HbA1c, hs-CRP, BUN, fasting glucose, fasting insulin, alanine aminotransferase, aspartate aminotransferase, gamma-glutamyl transpeptidase, and eGFR.

Categorical multivariate logistic regression was also employed to stratify subgroups based on gender, age, ethnicity, and BMI, to identify groups demonstrating significant results. Furthermore, smoothed curve fitting and saturation effect analysis investigated the nonlinear relationship between the RC/HDL-C ratio and HUA. After identifying nonlinearities, binary linear regression models were implemented on both sides of the threshold point. To assess the diagnostic efficacy of the RC/HDL-C ratio and other biochemical markers in patients with HUA, receiver operating characteristic (ROC) curve analysis was conducted. An outcome was deemed statistically significant if the *P* value was <.05.

## 3. Results

### 3.1. Study population demographic and clinical features

A total of 2795 participants were included in the study. Of these, 583 people were diagnosed with HUA. The average age of the participants was 45.80 ± 20.88 years. The overall population was 52.06% female and 47.94% male. The weighted distribution according to the HUA status classification is displayed in Table 1. Individuals diagnosed with HUA were more likely to be aged 60 years and over and male. Furthermore, the participants diagnosed with HUA exhibited considerably diminished levels of HDL-C, yet simultaneously exhibited significantly elevated levels of RC, BMI, TC, aspartate aminotransferase, TG, BUN, fasting plasma glucose, gamma-glutamyl transpeptidase, alanine aminotransferase, alkaline phosphatase, serum creatinine, LDL-C, SBP/DBP, HbA1c, hs-CRP, and SUA (*P* < .01). People without HUA had lower RC/HDL-C ratio levels than those with the condition (0.40 ± 0.31 vs 0.55 ± 0.35, *P* < .001).

**Table 1:** Weighted characteristics of participants with and without HUA status.

Characteristics	Non-HUA (n = 2212)	HUA (n = 583)	*P* value
Age (yr)	42.82 ± 18.60	50.18 ± 19.46	<.001
Age (%)			<.001
<40	46.11	34.02	
≥40, <60	32.69	28.08	
≥60	21.90	37.90	
Gender (%)			.011
Male	47.49	53.59	
Female	52.51	46.41	
Race (%)			.183
Mexican American	10.70	8.91	
Other Hispanic	6.96	7.28	
Non-Hispanic White	61.55	57.94	
Non-Hispanic Black	10.79	13.24	
Non-Hispanic Asian	5.32	6.50	
Other race	4.68	6.13	
Smoking behavior (%)			<.001
Never smoke	62.13	57.75	
Ever smoke	21.36	30.21	
Current smoke	16.51	12.04	
Heavy alcohol consumption (%)			.001
No	88.52	83.42	
Yes	11.48	16.58	
Hypertension (%)			<.001
No	67.28	36.43	
Yes	32.72	63.57	
T2DM (%)			<.001
No	87.97	79.60	
Yes	12.03	20.40	
Physical activity level (%)			.174
Inactive	46.65	48.31	
Moderate	8.06	10.09	
Active	45.29	41.60	
BMI (kg/m^2^)	28.00 ± 6.76	32.93 ± 8.30	<.001
BMI (%)			<.001
<25	36.48	13.98	
≥25, <30	31.03	29.69	
≥30	32.49	56.33	
SBP (mm Hg)	119.99 ± 17.18	127.40 ± 18.60	<.001
DBP (mm Hg)	70.97 ± 11.98	72.67 ± 13.02	.004
hs-CRP (mg/L)	1.47 (0.66–3.62)	2.74 (1.22–5.71)	<.001
FPG (mmol/L)	5.97 ± 1.64	6.21 ± 1.63	.002
HbA1c (%)	5.58 ± 0.85	5.77 ± 0.91	<.001
ALT (IU/L)	21.16 ± 16.30	26.34 ± 20.41	<.001
GGT (IU/L)	18.00 (13.00–28.00)	24.00 (17.00–38.00)	<.001
AST (IU/L)	21.46 ± 13.11	23.90 ± 13.32	<.001
TG (mg/dL)	111.86 ± 60.19	143.27 ± 70.62	<.001
TC (mg/dL)	182.23 ± 39.65	187.16 ± 42.52	.010
HDL-C (mg/dL)	55.10 ± 14.82	50.33 ± 14.88	<.001
LDL-C (mg/dL)	107.79 ± 34.35	111.61 ± 37.69	.024
RC (mg/dL)	19.33 ± 11.65	25.22 ± 13.76	<.001
SUA (μmol/L)	292.68 ± 61.00	438.42 ± 58.79	<.001
BUN (mg/dL)	13.73 ± 4.47	16.79 ± 6.71	<.001
Scr (mg/dL)	0.83 ± 0.29	0.96 ± 0.37	<.001
eGFR (mL/min/1.73 m^2^)	95.87 ± 64.35	94.31 ± 73.93	.731
RC/HDL-C	0.40 ± 0.31	0.55 ± 0.35	<.001

Mean ± SD or medians (quartile interval) were for continuous variables. Percent (%) was for categorical variables.

ALT = alanine aminotransferase, AST = aspartate aminotransferase, BMI = body mass index, BUN = blood urea nitrogen, DBP = diastolic blood pressure, eGFR = estimated glomerular filtration rate, FPG = fasting plasma glucose, GGT = gamma-glutamyl transpeptidase, HbA1c = glycosylated hemoglobin, HDL-C = high-density lipoprotein cholesterol, hs-CRP = high-sensitivity C-reactive protein, HUA = hyperuricemia, LDL-C = low-density lipoprotein cholesterol, RC = remnant cholesterol, RC/HDL-C = remnant cholesterol to high-density lipoprotein cholesterol ratio, SBP = systolic blood pressure, Scr = serum creatinine, SD = standard deviation, SUA = serum uric acid, T2DM = type 2 diabetes mellitus, TC = total cholesterol, TG = triglycerides.

### 3.2. Relationship between RC/HDL-C ratio and HUA

**Figure 2.**
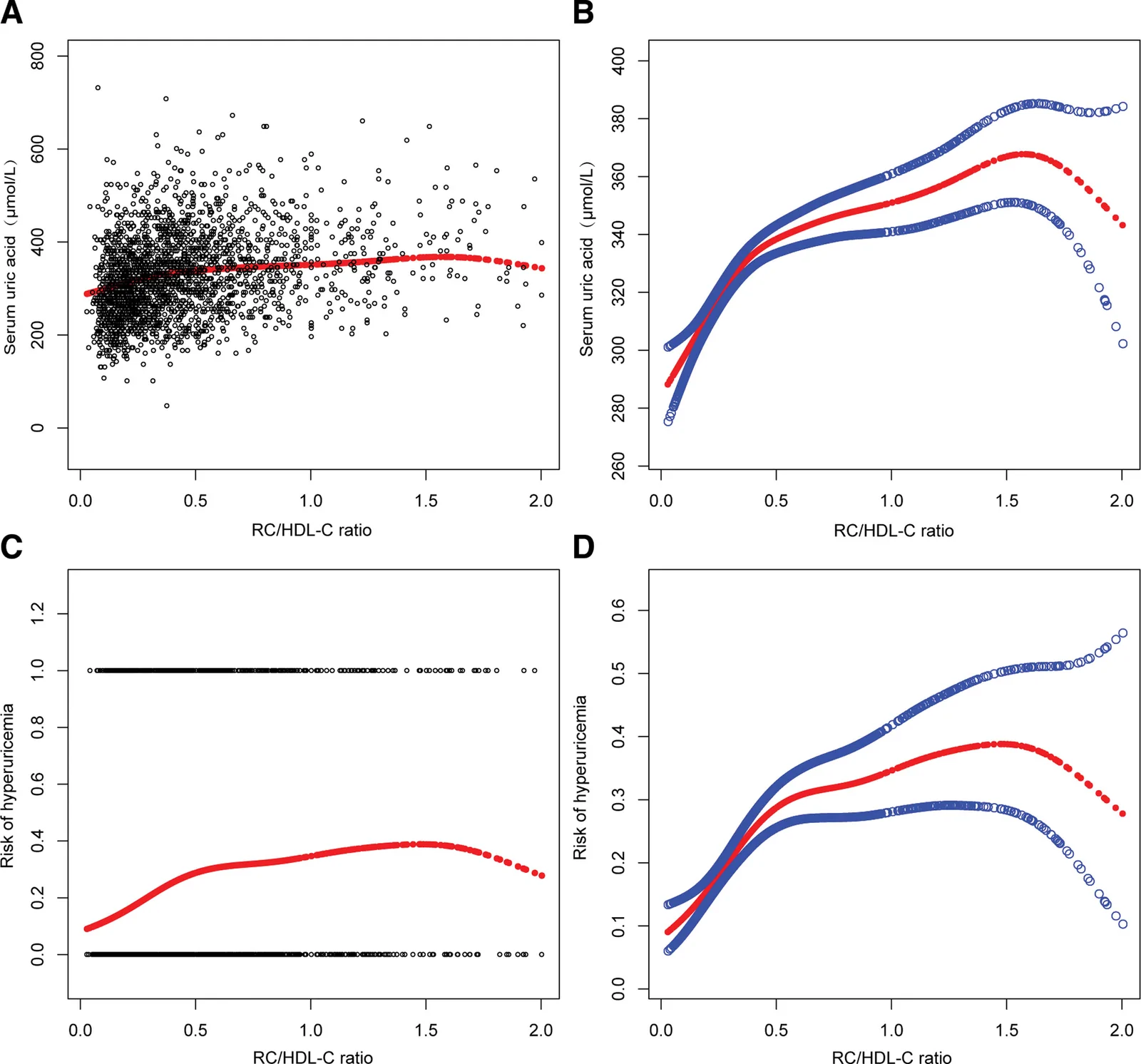
Associations between RC/HDL-C ratio, serum uric acid (SUA), and hyperuricemia (HUA) risk. (A, B) Associations between RC/HDL-C ratio and SUA. (C, D) Associations between RC/HDL-C ratio and risk of HUA. Each black point represents a sample. The solid red line represents the smooth curve fit between variables. Blue bands represent the 95% confidence interval from the fit. They were adjusted for age, gender, race, hypertension, BMI, T2DM, smoking, drinking, HbA1c, ALT, AST, GGT, hs-CRP, SBP, DBP, physical activity, eGFR, and BUN. ALT = alanine aminotransferase, AST = aspartate aminotransferase, BMI = body mass index, BUN = blood urea nitrogen, DBP = diastolic blood pressure, eGFR = estimated glomerular filtration rate, GGT = gamma-glutamyl transpeptidase, HbA1c = glycosylated hemoglobin, hs-CRP = high-sensitivity C-reactive protein, RC/HDL-C = remnant cholesterol to high-density lipoprotein cholesterol ratio, SBP = systolic blood pressure, T2DM = type 2 diabetes mellitus.

**Figure 3.**
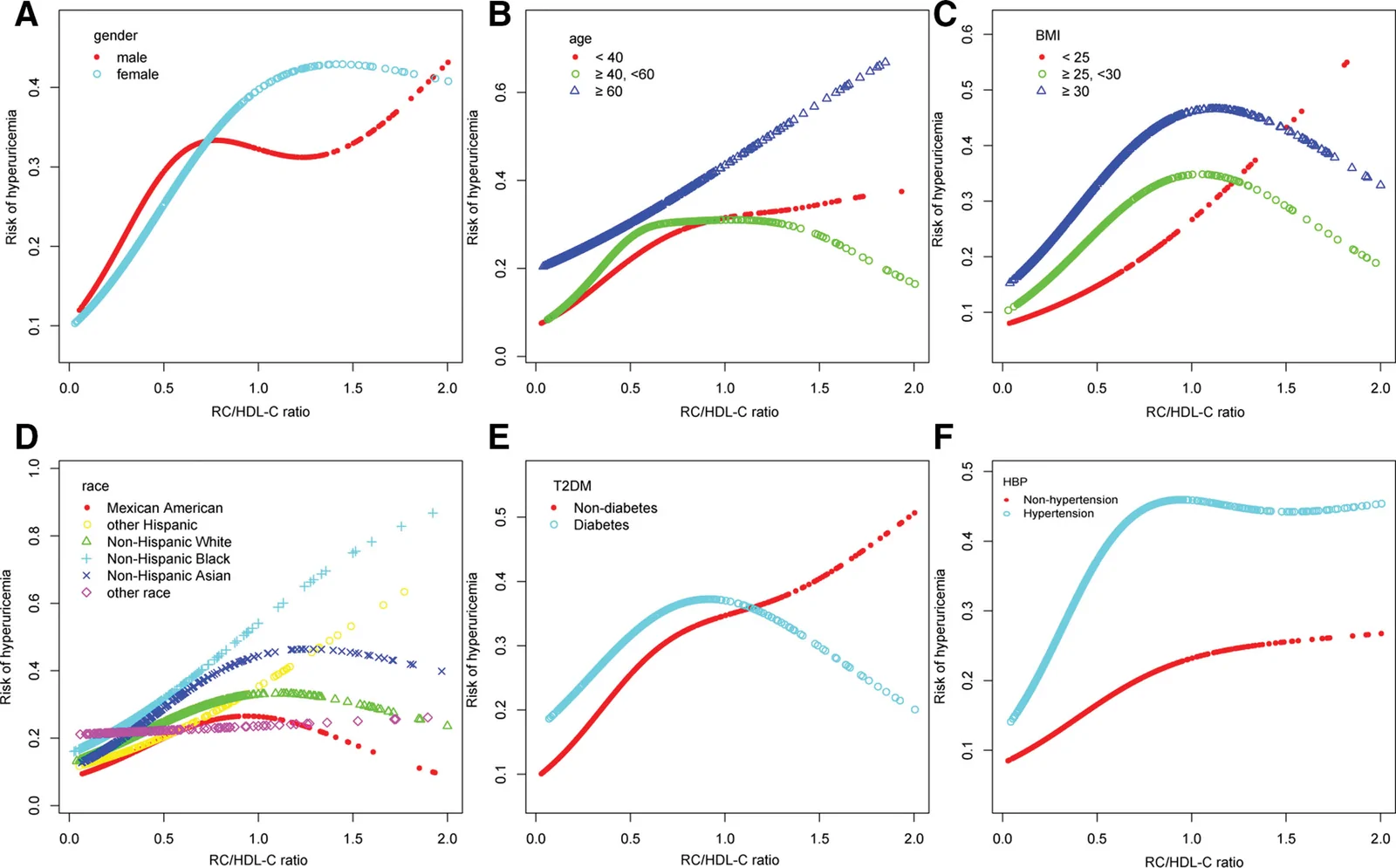
Associations between RC/HDL-C ratio and the risk of hyperuricemia (HUA) by gender (A), age (B), BMI (C), race (D), T2DM (E), and hypertension (F). They were adjusted for age, gender, race, hypertension, BMI, T2DM, smoking, drinking, HbA1c, ALT, AST, GGT, hs-CRP, SBP, DBP, physical activity, eGFR, and BUN. In subgroup analyses, the model was not adjusted for the classified variables. ALT = alanine aminotransferase, AST = aspartate aminotransferase, BMI = body mass index, BUN = blood urea nitrogen, DBP = diastolic blood pressure, eGFR = estimated glomerular filtration rate, GGT = gamma-glutamyl transpeptidase, HbA1c = glycosylated hemoglobin, hs-CRP = high-sensitivity C-reactive protein, RC/HDL-C = remnant cholesterol to high-density lipoprotein cholesterol ratio, SBP = systolic blood pressure, T2DM = type 2 diabetes mellitus.

To evaluate the relationship between the RC/HDL-C ratio and HUA prevalence, a multivariate regression analysis was performed, and the findings are shown in Table 2, Figure 2, and Figure 3. The findings of the multivariate logistic regression analysis showed that the levels of RC/HDL-C ratio in all 3 models were positively correlated with the prevalence of HUA: model 1 (odds ratio [OR] = 3.677, 95% confidence interval [CI]: 2.841, 4.760), model 2 (OR = 3.609, 95% CI: 2.739, 4.755), and model 3 (OR = 2.551, 95% CI: 1.834, 3.548). After converting continuous RC/HDL-C ratio values into categorical variables (quartiles), a significantly higher OR for developing HUA was observed in individuals in quartiles 2, 3, and 4 compared to those in quartile 1. The respective OR values were 52.7%, 163.2%, and 242.4%, as determined in model 3. Research findings indicate that individuals with elevated RC/HDL-C ratio levels are more susceptible to developing HUA than those with lower ratios.

**Table 2:** Associations between RC/HDL-C ratio and hyperuricemia status in different models.

	Model 1	Model 2	Model 3
	OR (95% CI), *P* value	OR (95% CI), *P* value	OR (95% CI), *P* value
RC/HDL-C ratio	3.677 (2.841, 4.760), <.001	3.609 (2.739, 4.755), <.001	2.551 (1.834, 3.548), .001
Q1 (0.026–0.197)	Reference	Reference	Reference
Q2 (0.197–0.329)	1.532 (1.112, 2.112), .009	1.502 (1.084, 2.081), .014	1.527 (1.034, 2.253), .033
Q3 (0.333–0.543)	2.870 (2.130, 3.866), <.001	2.686 (1.972, 3.658), <.001	2.632 (1.817, 3.812), <.001
Q4 (0.543–2.000)	4.354 (3.257, 5.820), <.001	4.302 (3.163, 5.851), <.001	3.425 (2.336, 5.020), <.001
* P* for trend	<.001	<.001	<.001
Subgroup analysis stratified by sex			
Men	2.872 (2.060, 4.006), <.001	2.969 (2.098, 4.201), <.001	2.073 (1.368, 3.142), <.001
Q1 (0.042–0.197)	Reference	Reference	Reference
Q2 (0.197–0.329)	1.464 (0.897, 2.387), .126	1.449 (0.885, 2.373), .140	1.374 (0.747, 2.530), .306
Q3 (0.333–0.543)	3.309 (2.126, 5.151), <.001	3.354 (2.134, 5.274), <.001	3.434 (1.974, 5.973), <.001
Q4 (0.543–2.000)	3.819 (2.479, 5.885), <.001	4.087 (2.606, 6.410), <.001	3.044 (1.730, 5.353), <.001
* P* for trend	<.001	<.001	<.001
Women	4.841 (3.181, 7.367), <.001	5.291 (3.350, 8.358), <.001	3.892 (2.226, 6.805), .029
Q1 (0.026–0.196)	Reference	Reference	Reference
Q2 (0.197–0.329)	1.566 (1.023, 2.397), .039	1.607 (1.035, 2.494), .034	1.737 (1.021, 2.956), .041
Q3 (0.333–0.543)	2.294 (1.516, 3.471), <.001	2.059 (1.333, 3.181), <.001	1.765 (1.031, 3.023), .038
Q4 (0.543–2.000)	4.851 (3.250, 7.239), <.001	5.086 (3.308, 7.818), <.001	4.592 (2.625, 8.033), <.001
* P* for trend	<.001	<.001	<.001
Subgroup analysis stratified by age (yr)			
* *<40	5.412 (3.385, 8.652), <.001	4.896 (3.006, 7.973), <.001	2.380 (1.263, 4.484), .007
* *≥40, <60	2.380 (1.527, 3.710), <.001	2.696 (1.665, 4.367), <.001	2.415 (1.347, 4.328), .003
* *≥60	3.394 (2.141, 5.378), <.001	4.239 (2.600, 6.911), <.001	3.281 (1.840, 5.852), <.001
Subgroup analysis stratified by BMI (Kg/m^2^)			
* *<25	4.823 (2.490, 9.340), <.001	3.059 (1.503, 6.225), .002	3.803 (1.575, 9.185), .002
* *≥25, <30	2.405 (1.471, 3.931), <.001	2.252 (1.328, 3.818), .008	2.360 (1.244, 4.478), .002
* *≥30	2.516 (1.751, 3.615), <.001	2.666 (1.800, 3.949), <.001	2.415 (1.550, 3.764), <.001
Subgroup analysis stratified by race			
Mexican American	2.626 (1.340, 5.148), .004	2.181 (1.088, 4.374), .02	1.884 (0.768, 4.625), .166
Other Hispanic	5.574 (2.236, 13.893), <.001	4.273 (1.652, 11.051), .002	5.954 (1.634, 21.692), .006
Non-Hispanic White	3.871 (2.469, 6.067), <.001	3.584 (2.244, 5.725), <.001	2.360 (1.321, 4.217), .003
Non-Hispanic Black	12.543 (5.609, 28.049), <.001	9.961 (4.422, 22.438), <.001	5.367 (2.074, 13.889), <.001
Non-Hispanic Asian	4.102 (2.183, 7.706), <.001	3.676 (1.917, 7.050), <.001	2.122 (0.879, 5.122), .094
Other race	2.256 (0.879, 5.791), .090	2.016 (0.764, 5.320), .156	1.268 (0.280, 5.742), .758
Subgroup analysis stratified by T2DM			
No	4.048 (2.961, 5.534), <.001	3.985 (2.853, 5.567), <.001	2.980 (2.013, 4.412), .019
Yes	1.944 (1.190, 3.178), .007	2.744 (1.606, 4.689), <.001	1.952 (1.040, 3.664), .003
Subgroup analysis stratified by hypertension			
No	3.418 (2.259, 5.171), <.001	3.017 (1.929, 4.718), <.001	2.155 (1.268, 3.663), .004
Yes	2.862 (2.021, 4.052), <.001	3.454 (2.376, 5.022), <.001	2.804 (1.833, 4.288), <.001

Model 1: no covariates were adjusted. Model 2: age, gender, and race were adjusted. Model 3: age, gender, race, hypertension, BMI, T2DM, smoking status, drinking status, HbA1c, ALT, AST, GGT, hs-CRP, SBP, DBP, physical activity, eGFR, and BUN were adjusted. In the subgroup analysis, the grouping indicators were not subject to adjustment within the model.

ALT = alanine aminotransferase, AST = aspartate aminotransferase, BMI = body mass index, BUN = blood urea nitrogen, CI = confidence interval, DBP = diastolic blood pressure, eGFR = estimated glomerular filtration rate, GGT = gamma-glutamyl transpeptidase, HbA1c = glycosylated hemoglobin, hs-CRP = high-sensitivity C-reactive protein, OR = odds ratios, RC/HDL-C = remnant cholesterol to high-density lipoprotein cholesterol ratio, SBP = systolic blood pressure, T2DM = type 2 diabetes mellitus.

Gender-stratified subgroup analyses across all 3 models indicate a positive correlation between male HUA risk and RC/HDL-C ratio. This was observed in model 1 (OR = 2.872, 95% CI: 2.060, 4.006), model 2 (OR = 2.969, 95% CI: 2.098, 4.201), and model 3 (OR = 2.073, 95% CI: 1.368, 3.142). Similarly consistent findings were observed for women, with all 3 models demonstrating a positive correlation between HUA and the RC/HDL-C ratio: model 1 (OR = 4.841, 95% CI: 3.181, 7.367), model 2 (OR = 5.291, 95% CI: 3.350, 8.358), and model 3 (OR = 3.892, 95% CI: 2.226, 6.805). Subgroup analysis by age cohort revealed that adults with elevated RC/HDL-C ratio levels exhibited an increased risk of developing HUA, particularly those aged 60 years and above, compared with individuals in other age groups. This finding was consistently observed across all 3 models: model 1 (OR = 3.394, 95% CI: 2.141, 5.378), model 2 (OR = 4.239, 95% CI: 2.600, 6.911), and model 3 (OR = 3.281, 95% CI: 1.840, 5.852). People with a BMI of <25 kg/m^2^ may be more likely to develop HUA than those in other BMI categories, which was supported by the findings of model 1 (OR = 4.823, 95% CI: 2.490, 9.340), and in models 2 and 3, the OR value was 3.059 (95% CI: 1.503, 6.225) and 3.803 (95% CI: 1.575, 9.185), respectively. As demonstrated in models 1, 2, and 3, elevated RC/HDL-C ratio levels were associated with a significant increase in the risk of HUA among Other Hispanic populations. The OR for model 1 was 5.574 (95% CI: 2.236, 13.893), for model 2, was 4.273 (95% CI: 1.652, 11.051), and for model 3, was 5.954 (95% CI: 1.634, 21.692).

### 3.3. Association between RC/HDL-C ratio and SUA level

**Figure 4.**
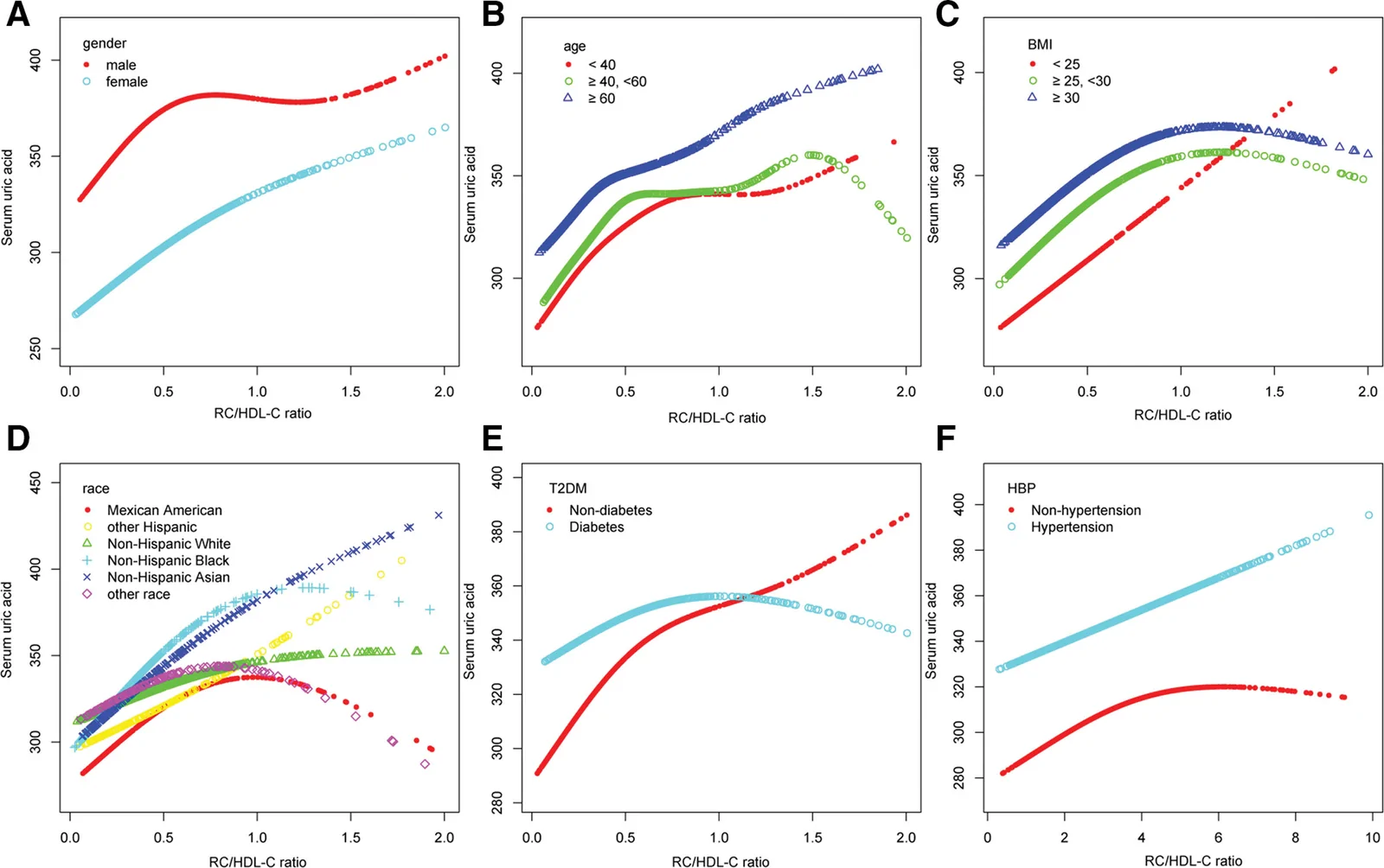
Associations between RC/HDL-C ratio and serum uric acid (SUA) by gender (A), age (B), BMI (C), race (D), T2DM (E), and hypertension (F). They were adjusted for age, gender, race, hypertension, BMI, T2DM, smoking, drinking, HbA1c, ALT, AST, GGT, hs-CRP, SBP, DBP, physical activity, eGFR, and BUN. In subgroup analyses, the model was not adjusted for the classified variables. ALT = alanine aminotransferase, AST = aspartate aminotransferase, BMI = body mass index, BUN = blood urea nitrogen, DBP = diastolic blood pressure, eGFR = estimated glomerular filtration rate, GGT = gamma-glutamyl transpeptidase, HbA1c = glycosylated hemoglobin, hs-CRP = high-sensitivity C-reactive protein, RC/HDL-C = remnant cholesterol to high-density lipoprotein cholesterol ratio, SBP = systolic blood pressure, T2DM = type 2 diabetes mellitus.

As shown in Table 3, Figure 2, and Figure 4, a significant correlation exists between the RC/HDL-C ratio and SUA levels. The regression model indicates a strong positive association between SUA levels and RC/HDL-C ratio, with a *P* value <.001. This was observed in models 1 (β = 76.901, 95% CI: 67.264, 86.538), models 2 (β = 58.916, 95% CI: 49.786, 68.047), and models 3 (β = 42.963, 95% CI: 33.226, 52.699). Furthermore, compared with the lower quartile, SUA levels exhibited a significant upward trend with increasing quartiles of the RC/HDL-C ratio (trend *P* < .001). Simultaneously, compared with Q1, the adjusted mean differences (β) for Q2, Q3, and Q4 increased by 16.455 dB/m (95% CI: 7.821, 25.088), 32.452 dB/m (95% CI: 23.569, 41.335), and 44.851 dB/m (95% CI: 35.448, 54.254), respectively. After adjusting for all relevant factors, a significant correlation was observed between SUA levels and RC/HDL-C ratio in both male (β = 37.032, 95% CI: 23.707, 50.358) and female (β = 51.365, 95% CI: 37.128, 65.602) subjects. Moreover, subgroup analyses by age and BMI revealed significant correlations between RC/HDL-C ratio and SUA levels, particularly in individuals aged 60 years and above (β = 53.142, 95% CI: 34.391, 71.894) and among individuals with a BMI below 25 kg/m^2^ (β = 59.468, 95% CI: 38.412, 80.524).

**Table 3:** Correlation between RC/HDL-C ratio and SUA (μmol/L) in different models.

	Model 1	Model 2	Model 3
	β (95% CI), *P* value	β (95% CI), *P* value	β (95% CI), *P* value
RC/HDL-C (per SD increase)	76.901 (67.264, 86.538), <.001	58.916 (49.786, 68.047), <.001	42.963 (33.226, 52.699), <.001
Q1 (0.026–0.197)	Reference	Reference	Reference
Q2 (0.197–0.329)	20.121 (11.349, 28.894), <.001	15.974 (7.979, 23.970), <.001	16.455 (7.821, 25.088), <.001
Q3 (0.333–0.543)	46.904 (38.153, 55.654), <.001	36.216 (28.042, 44.389), <.001	32.452 (23.569, 41.335), <.001
Q4 (0.543–2.000)	69.810 (61.050, 78.570), <.001	55.039 (46.680, 63.399), <.001	44.851 (35.448, 54.254), <.001
* P* for trend	<.001	<.001	<.001
Subgroup analysis stratified by sex			
Men	51.932 (39.985, 63.879), <.001	52.268 (39.973, 64.564), <.001	37.032 (23.707, 50.358), <.001
Women	73.444 (59.677, 87.212), <.001	69.786 (56.170, 83.401), <.001	51.365 (37.128, 65.602), <.001
Subgroup analysis stratified by age (yr)			
<40	84.340 (68.990, 99.689), <.001	65.118 (51.516, 78.721), <.001	33.809 (17.390, 50.229), <.001
≥40, <60	61.846 (45.042, 78.651), <.001	46.792 (31.291, 62.293), <.001	40.452 (24.766, 56.139), <.001
≥60	72.680 (54.204, 91.156), <.001	71.815 (53.236, 90.394), <.001	53.142 (34.391, 71.894), <.001
Subgroup analysis stratified by BMI (kg/m^2^)			
<25	83.193 (63.227, 103.159), <.001	49.550 (31.171, 67.929), <.001	59.468 (38.412, 80.524), <.001
≥25, <30	60.329 (41.977, 78.682), <.001	38.326 (21.533, 55.119), <.001	43.950 (27.058, 60.842), <.001
≥30	55.609 (40.855, 70.362), <.001	41.076 (26.581, 55.571), <.001	40.686 (25.247, 56.126), <.001
Subgroup analysis stratified by race			
Mexican American	61.001 (38.035, 83.967), <.001	44.013 (23.866, 64.161), <.001	31.862 (8.659, 55.065), .007
Other Hispanic	82.351 (49.895, 114.806), <.001	59.330 (29.360, 89.300), <.001	65.449 (32.028, 98.869), <.001
Non-Hispanic White	72.839 (56.555, 89.123), <.001	50.841 (35.266, 66.417), <.001	32.682 (16.166, 49.198), <.001
Non-Hispanic Black	134.235 (105.854, 162.615), <.001	101.176 (74.739, 127.613), <.001	67.684 (38.903, 96.465), <.001
Non-Hispanic Asian	99.469 (77.398, 121.540), <.001	76.205 (55.757, 96.654), <.001	56.878 (35.208, 78.548), <.001
Other race	43.814 (10.191, 77.437), .011	25.078 (−7.120, 57.276), .128	11.265 (−26.250, 48.780), .557
Subgroup analysis stratified by T2DM			
No	83.958 (72.821, 95.095), <.001	64.070 (53.700, 74.441), <.001	47.987 (36.997, 58.977), <.001
Yes	40.410 (18.811, 62.010), <.001	45.268 (23.877, 66.659), <.001	25.361 (4.074, 46.649), .019
Subgroup analysis stratified by hypertension			
No	70.783 (58.089, 83.477), <.001	51.749 (40.179, 63.319), <.001	38.692 (26.054, 51.329), <.001
Yes	65.619 (51.027, 80.212), <.001	60.046 (45.427, 74.665), <.001	45.105 (30.671, 59.539), <.001

Model 1: no covariates were adjusted. Model 2: age, gender, and race were adjusted. Model 3: age, gender, race, hypertension, BMI, T2DM, smoking status, drinking status, HbA1c, ALT, AST, GGT, hs-CRP, SBP, DBP, physical activity, eGFR, and BUN were adjusted. In the subgroup analysis, the grouping indicators were not subject to adjustment within the model.

β = standardized regression coefficient, ALT = alanine aminotransferase, AST = aspartate aminotransferase, BMI = body mass index, BUN = blood urea nitrogen, CI = confidence interval, DBP = diastolic blood pressure, eGFR = estimated glomerular filtration rate, GGT = gamma-glutamyl transpeptidase, HbA1c = glycosylated hemoglobin, hs-CRP = high-sensitivity C-reactive protein, RC/HDL-C = remnant cholesterol to high-density lipoprotein cholesterol ratio, SBP = systolic blood pressure, SD = standard deviation, SUA = serum uric acid, T2DM = type 2 diabetes mellitus.

### 3.4. Nonlinear relationship analysis

The relationship between the RC/HDL-C ratio and the risk of HUA in the general population exhibited an inverted U-shaped curve (Fig. 2). Further subgroup analyses, categorized according to age, ethnicity, gender, BMI, hypertension status, and T2DM status, indicated a nonlinear relationship between the risk of HUA and RC/HDL-C ratio (Table 4 and Fig. 3). The RC/HDL-C ratio inflection point for the overall population HUA risk was 0.400. Subgroup analyses revealed inflection points of 0.698, 0.481, 1.067, and 0.415 for women, aged 40 to 59 years, Mexican Americans, and individuals with a BMI between 25 and 30 kg/m^2^. Furthermore, it was observed that in the subgroups of patients with hypertension and T2DM, the RC/HDL-C ratio inflection points on the risk curve for HUA were 0.415 and 0.392, respectively.

**Table 4:** Threshold effect analysis of RC/HDL-C ratio on the prevalence of HUA in different genders, ages, BMI, race, T2DM, and hypertension using the two-piecewise linear regression model.

Prevalence of HUA	Adjusted OR (95% CI), *P* value
All participants	
Fitting by the standard linear model	2.551 (1.834, 3.548), <.001
Fitting by the two-piecewise linear model	
Inflection point	0.400
RC/HDL-C < 0.400	59.153 (15.074, 232.122), <.001
RC/HDL-C > 0.400	1.423 (0.943, 2.147), .092
Log likelihood ratio	<0.001
Women	
Fitting by the standard linear model	3.892 (2.226, 6.805), <.001
Fitting by the two-piecewise linear model	
Inflection point	0.698
RC/HDL-C < 0.698	12.575 (4.395, 35.976), <.001
RC/HDL-C > 0.698	1.115 (0.362, 3.433), .849
Log likelihood ratio	0.009
Age 40–59 (yr)	
Fitting by the standard linear model	2.415 (1.347, 4.328), .003
Fitting by the two-piecewise linear model	
Inflection point	0.481
RC/HDL-C < 0.481	256.596 (28.361, 2321.534), <.001
RC/HDL-C > 0.481	0.753 (0.339, 1.672), .485
Log likelihood ratio	<0.001
BMI ≥ 25, <30 (Kg/m^2^)	
Fitting by the standard linear model	2.360 (1.244, 4.478), .009
Fitting by the two-piecewise linear model	
Inflection point	0.415
RC/HDL-C < 0.415	220.957 (18.100, 2697.338), <.001
RC/HDL-C > 0.415	0.884 (0.387, 2.022), .770
Log likelihood ratio	<0.001
Mexican American	
Fitting by the standard linear model	1.884 (0.768, 4.625), .166
Fitting by the two-piecewise linear model	
Inflection point	1.067
RC/HDL-C < 1.067	6.503 (1.694, 24.962), .006
RC/HDL-C > 1.067	0.034 (0.001, 1.422), .076
Log likelihood ratio	0.013
T2DM	
Fitting by the standard linear model	1.952 (1.040, 3.664), .0374
Fitting by the two-piecewise linear model	
Inflection point	0.392
RC/HDL-C < 0.392	380.012 (11.920, 12,114.806), <.001
RC/HDL-C > 0.392	1.042 (0.493, 2.206), .9137
Log likelihood ratio	0.001
Hypertension	
Fitting by the standard linear model	2.804 (1.833, 4.288), <.001
Fitting by the two-piecewise linear model	
Inflection point	0.415
RC/HDL-C < 0.415	109.710 (20.820, 578.099), <.001
RC/HDL-C > 0.415	1.387 (0.826, 2.329), .2155
Log likelihood ratio	<0.001

Age, gender, race, hypertension, BMI, T2DM, smoking status, drinking status, HbA1c, ALT, AST, GGT, hs-CRP, SBP, DBP, physical activity, eGFR, and BUN were adjusted. In the subgroup analysis, the grouping indicators were not subject to adjustment within the model.

ALT = alanine aminotransferase, AST = aspartate aminotransferase, BMI = body mass index, BUN = blood urea nitrogen, CI = confidence interval, DBP = diastolic blood pressure, eGFR = estimated glomerular filtration rate, GGT = gamma-glutamyl transpeptidase, HbA1c = glycosylated hemoglobin, hs-CRP = high-sensitivity C-reactive protein, HUA = hyperuricemia, OR= odds ratio, RC/HDL-C = remnant cholesterol to high-density lipoprotein cholesterol ratio, SBP = systolic blood pressure, T2DM = type 2 diabetes mellitus.

### 3.5. A ROC analysis of RC/HDL-C ratio as a HUA predictor

The ROC curve analysis was used to evaluate the diagnostic effectiveness of the RC/HDL-C ratio for HUA. The results showed that the area under the curve (AUC) for the RC/HDL-C ratio was 0.654 (95% CI: 0.630, 0.679), as shown in Table S1, Supplemental Digital Content, https://links.lww.com/MD/R676 and Figure S1, Supplemental Digital Content, https://links.lww.com/MD/R677. The RC/HDL-C ratio demonstrated superior diagnostic accuracy for HUA due to its higher AUC value than other biomarkers, such as HDL-C and RC. Furthermore, subgroup analyses of the ROC curve by gender also demonstrated that the AUC value for the RC/HDL-C ratio was higher than that for other biomarkers (Fig. S2, Supplemental Digital Content, https://links.lww.com/MD/R677). As shown in Table S1, Supplemental Digital Content, https://links.lww.com/MD/R676, the AUC for the RC/HDL-C ratio was 0.657 (95% CI: 0.620, 0.694) in women and 0.638 (95% CI: 0.605, 0.672) in men.

## 4. Discussion

To the best of our knowledge, our study provides a comprehensive assessment of the association between the RC/HDL-C ratio, HUA, and SUA levels, using data from a national cross-sectional survey conducted in the United States between 2017 and 2018. The RC/HDL-C ratio showed a strong positive correlation with the risk of developing HUA within the study, particularly among females, individuals over 60 years, Other Hispanic ethnicities, and those with a BMI below 25 kg/m^2^. The inflection point for the diagnostic threshold for RC/HDL-C levels in predicting HUA risk across the general population was 0.400. This shows that when the RC/HDL-C ratio level is lower than 0.400, the risk of HUA is positively correlated with the RC/HDL-C ratio. ROC analysis showed that the RC/HDL-C ratio was more effective than RC or HDL-C alone in identifying HUA.

Research into the relationship between the RC/HDL-C ratio and disease is limited. Most studies have focused on investigating whether an elevated RC/HDL-C ratio is associated with an increased risk of developing T2DM, coronary heart disease, nonalcoholic fatty liver disease, MetS, atherosclerosis, and sarcopenia, and these studies have shown that the RC/HDL-C ratio is a valuable indicator of metabolic disease risk.^[30–34]^ Recently, Li et al’s cross-sectional study focused on newly diagnosed diabetes, incorporating data from 100,309 Chinese adults with normal baseline blood glucose levels. The findings revealed a positive correlation between the RC/HDL-C ratio and prediabetes risk.^[35]^ Zou et al previously conducted a second cohort study named Nonalcoholic Fatty Liver Disease in the Gifu Area Longitudinal Analysis, which involved 14,251 Japanese adults undergoing health examinations. Their findings revealed that elevated RC/HDL-C ratios were strongly associated with an increased risk of nonalcoholic fatty liver disease.^[31]^ A prospective cohort study was recently conducted by Tan et al, involving 84,380 participants, with a median follow-up duration of 16.92 years. The study revealed that RC/HDL-C levels significantly influenced the occurrence of atherosclerotic cardiovascular disease.^[36]^ It is well established that HUA is closely associated with inflammation, IR, and lipid toxicity.^[35]^ HUA often occurs alongside conditions such as T2DM, atherosclerotic cardiovascular disease, hyperlipidemia, hypertension, and obesity. Concurrently, HUA is a significant independent predictor of MetS. The risk of MetS increases with rising SUA levels.^[37]^ Evidence shows that partially overlapping pathophysiological mechanisms underlie the association between HUA and these diseases.^[38–40]^ Our findings indicate that HUA strongly correlates with RC/HDL-C, similar to the abovementioned diseases. Concurrently, our large-scale subgroup analysis across multiethnic populations in the United States effectively validated the stability and sensitivity of these findings. Based on these discoveries, our study demonstrates a significant correlation between the RC/HDL-C ratio and the HUA and SUA levels.

No definitive mechanism has yet been established that links the risk of HUA to an elevated RC/HDL-C ratio. RC is a TG-rich lipoprotein, while HDL-C concentration in the plasma negatively correlates with TG levels. This suggests a bidirectional regulatory relationship between these lipid parameters.^[41]^ Based on prior research, we hypothesize that the following factors are the most probable underlying mechanisms. First, due to their small size, RC particles are directly phagocytosed by macrophages, forming foam cells that deposit on the renal arteries’ intima. This can lead to vascular stenosis or occlusion, precipitating renal arteriosclerosis. This condition further impairs renal function, reducing UA excretion and consequently inducing HUA.^[42,43]^ Second, RC has unique pro-inflammatory properties and can induce endoplasmic reticulum stress and mitochondrial dysfunction. This promotes the production of reactive oxygen species.^[44,45]^ Furthermore, elevated RC levels correlate with persistent low-grade systemic inflammation and increased inflammatory markers, such as hs-CRP, in the blood. Consequently, RC promotes the progression of chronic kidney disease.^[41,46]^ Finally, IR is a factor closely associated with the pathogenesis of HUA.^[47]^ It enhances UA reabsorption by stimulating the urate transporter URAT1 and/or the non-NAD-dependent anion cotransporter in the brush border membrane of the proximal renal tubule.^[48]^ IR may also contribute to the relationship between lipids and HUA through inflammation and endoplasmic reticulum stress.^[49]^

Conversely, the physiological effects of HDL-C are beneficial. HDL-C is a key regulator of lipid metabolism and has also been demonstrated to possess anti-inflammatory and antioxidant properties. HDL-C improves endothelial function by suppressing inflammatory responses in endothelial cells and reducing the expression of key cell adhesion molecules.^[50]^ The primary component of HDL-C, apolipoprotein A-1, exerts its anti-inflammatory effects by inhibiting the activation of CD11b on monocytes.^[51]^On the other hand, a negative correlation exists between HDL-C levels and renal function. Reduced HDL-C levels indicate a decline in eGFR, which may impair renal clearance of UA. Moreover, HDL-C concentrations exhibit a significant negative correlation with IR, which impairs UA metabolism and contributes to HUA. These factors interact synergistically, collectively promoting the onset and progression of HUA. Therefore, further research is required to elucidate the precise mechanisms underlying the relationship between RC/HDL-C and HUA risk.

A notable finding of the study was that, following a subgroup analysis by gender, women exhibited a higher risk of developing HUA than men as RC/HDL-C levels increased. This outcome had not been previously documented in extant research. It is hypothesized that this outcome may be associated with the age distribution within the female cohort, given that the mean age of women in this study was 44.91 ± 19.32 years, with the vast majority being postmenopausal or perimenopausal. Following the cessation of menstruation, estrogen levels in women undergo a substantial decline. Estrogen exerts a dual effect on UA production. First, it inhibits xanthine oxidase activity and decreases activation of the pentose phosphate pathway, thereby reducing the rate of UA production. Second, estrogen promotes UA excretion by upregulating the expression of UA transporters.^[52,53]^ Moreover, existing literature indicates that estrogen significantly reduces inflammation and enhances insulin sensitivity via the estrogen receptor alpha signaling pathway. However, the decline in estrogen levels following menopause has been demonstrated to exacerbate IR and disrupt UA metabolism, thereby increasing women’s risk of developing HUA.^[54,55]^ Consequently, fluctuations in estrogen levels may be a primary sex hormone-related cause of HUA in postmenopausal women. Future research endeavors must encompass fundamental investigations and large-scale, multicentre longitudinal clinical studies. Therefore, the change of estrogen level may be the primary sex hormone cause of postmenopausal HUA. In the future, we need to do more basic and longitudinal clinical research based on large samples and multiple people to explore the mechanism.

A notable finding was the stronger association between the RC/HDL-C ratio and HUA risk in individuals with BMI < 25kg/m^2^ compared to overweight/obese individuals. This appears inconsistent with the well-documented link between obesity and HUA, but several mechanisms may explain this observation. First, in obese populations, HUA risk is driven by multifactorial pathways, including adipose tissue inflammation, IR-mediated urate reabsorption, and renal fat accumulation, which may mask the independent effect of lipid imbalance. By contrast, nonobese individuals lack these obesity-related confounders, making the RC/HDL-C ratio a more sensitive marker of HUA risk. Second, RC can directly induce renal arteriosclerosis via foam cell formation; in nonobese individuals with no preexisting renal impairment, this RC-mediated damage has a more direct impact on urate excretion.^[23,42]^ Third, HDL-C’s protective role against HUA may be more compromised in nonobese individuals with elevated RC: nonobese populations typically have higher baseline HDL-C, so RC-induced TG-cholesterol exchange and subsequent HDL-C dysfunction would result in a more pronounced loss of protection. Reanalysis with additional confounders confirmed the robustness of this association, suggesting that the RC/HDL-C ratio may serve as a critical HUA screening tool – especially in nonobese individuals, who are often overlooked in HUA risk assessment due to the perception that obesity is the primary driver.

The primary strength of this study lies in its large, nationally representative sample size, enabling a preliminary investigation into the relationship between the RC/HDL-C ratio and the risk of developing HUA and SUA levels. Furthermore, sensitivity analyses were conducted across different population subgroups to validate the robustness of the findings. Through curve fitting, the study identified that within an appropriate range (<0.400), the RC/HDL-C ratio may assist in diagnosing HUA. Nevertheless, this study has several limitations. First, as the database is a cross-sectional survey, it is difficult to establish a definitive causal relationship between HUA and the RC/HDL-C ratio. Second, recall bias may exist in some self-reported questionnaires, potentially leading to biased results. Furthermore, although several relevant covariates were adjusted for in our analysis, the study is subject to potential confounding from unmeasured or unavailable variables. Specifically, data on kidney injury, gout, genetic purine metabolism disorders, and specific medications were not fully available in the database, precluding their formal exclusion or adjustment. These factors may influence SUA and lipid levels and thus could affect the observed associations. Additionally, dietary habits and lifestyle factors, which were not comprehensively captured in the dataset, may also contribute to residual confounding. Finally, as all study participants were American, future prospective, multiethnic, multicentre studies are required to validate the external applicability of this research and address the limitations identified in prior investigations.

## 5. Conclusion

In the American population, an increase in the RC/HDL-C ratio is independently related to an increased risk of HUA and higher SUA levels. This correlation remains stable when further stratified by gender, age, and BMI. The RC/HDL-C ratio may be a simple, effective, noninvasive diagnostic tool for identifying HUA.

## Acknowledgments

The authors thank the staff and the participants of the NHANES study for their valuable contributions.

## Author contributions

**Conceptualization:** Yanyan Xuan, Qi Yao.

**Funding acquisition:** Yanyan Xuan.

**Methodology:** Jinbo Yu.

**Formal analysis:** Yanyan Xuan, Haibo Chen.

**Data curation:** Yuanyuan Cen.

**Investigation:** Jinbo Yu, Haibo Chen.

**Software:** Fangfang He.

**Supervision:** Qi Yao.

**Writing – original draft:** Yanyan Xuan, Fangfang He, Haibo Chen.

**Writing – review & editing:** Yanyan Xuan, Yuanyuan Cen.

## Supplementary Material




