# Supplementary material for: Nonlinear relationship between the ratio of remnant cholesterol to high-density lipoprotein cholesterol and hyperuricemia in the general population
Source: Medicine (Baltimore). 2026 Apr 10;105(15):e48360. doi: 10.1097/MD.0000000000048360 (PMC13593280; doi:10.1097/MD.0000000000048360)
Supplement: Supplementary file 1 [file medi-105-e48360-s001.pdf]

Supplementary Material

Supplemental Table 1. The best threshold, sensitivities, specificities, and AUC of each parameter for screening HUA in the general population and subgroup analysis for gender.

	AUC	95% CI	Best threshold	Specificity	Sensitivity
RC/HDL-C	0.6541	0.6296-0.6785	0.3583	0.5954	0.6621
RC	0.6391*	0.6147-0.6635	16.5000	0.5226	0.7015
HDL-C	0.6251*	0.5994-0.6507	48.5000	0.6121	0.5901
**Subgroup analysis stratified by sex**					
**Men**					
RC/HDL-C	0.6383	0.6050-0.6716	0.3587	0.5313	0.7170
RC	0.6198*	0.5862-0.6534	16.5000	0.4922	0.7233
HDL-C	0.6280*	0.5931-0.6628	46.5000	0.5724	0.6415
**Women**					
RC/HDL-C	0.6570	0.6200-0.6940	0.3483	0.6412	0.6075
RC	0.6535*	0.6175-0.6896	17.5000	0.5916	0.6528
HDL-C	0.6033*	0.5648-0.6419	53.5000	0.5874	0.5887

* $P < 0.01$, compared with RC/HDL-C ratio. **Abbreviations:** ROC, Receiver operating characteristic; AUC, Area under curve.
