# Supplementary material for: Nonlinear relationship between the ratio of remnant cholesterol to high-density lipoprotein cholesterol and hyperuricemia in the general population
Source: Medicine (Baltimore). 2026 Apr 10;105(15):e48360. doi: 10.1097/MD.0000000000048360 (PMC13593280; doi:10.1097/MD.0000000000048360)
Supplement: Supplementary file 2 [file medi-105-e48360-s002.pdf]

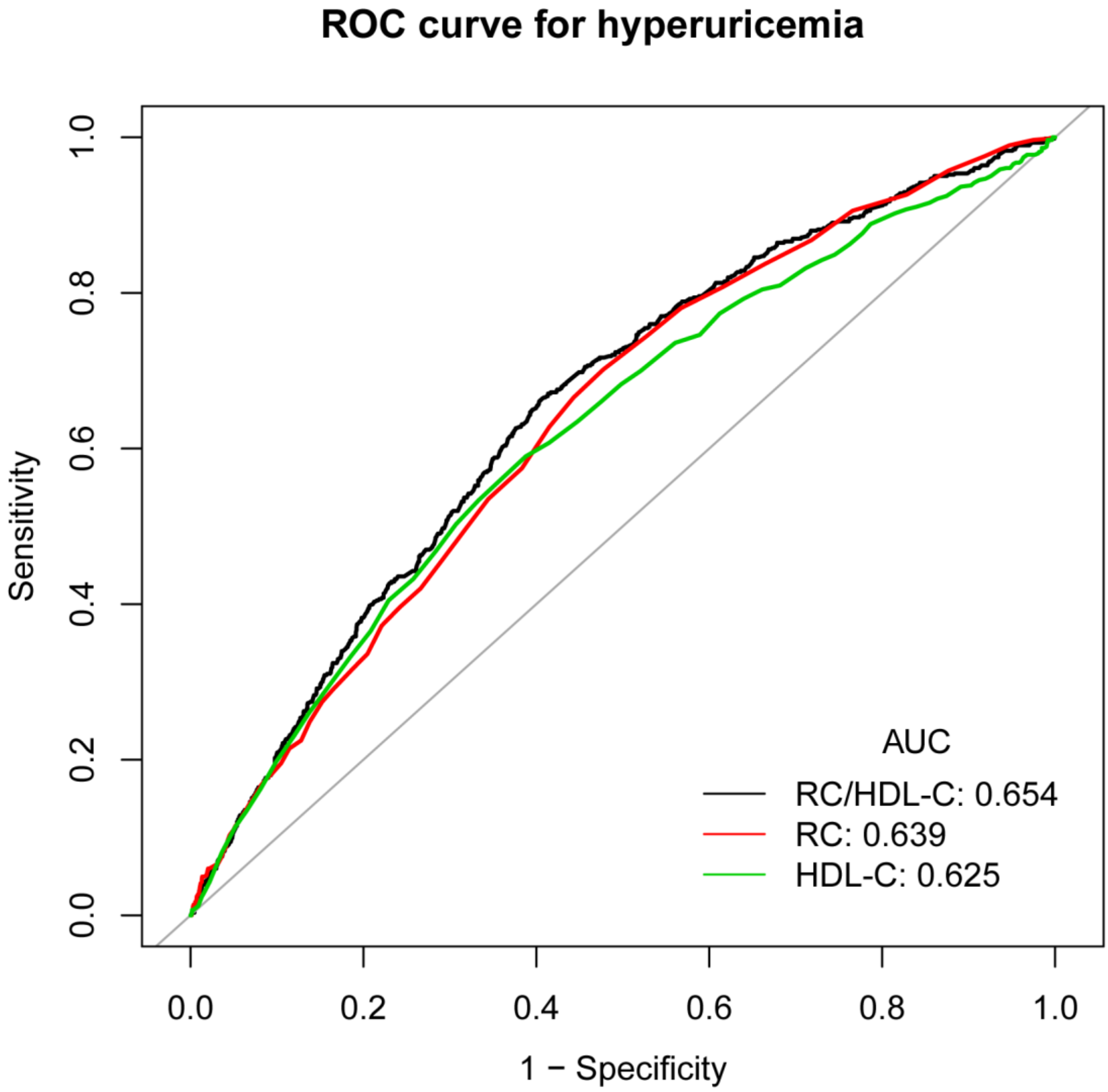

Supplementary Figure 1. ROC curves for RC/HDL-C ratio, compared to HDL-C and RC for HUA onset. As determined by AUC, the predictive value for the RC/HDL-C ratio is more significant than other factors.

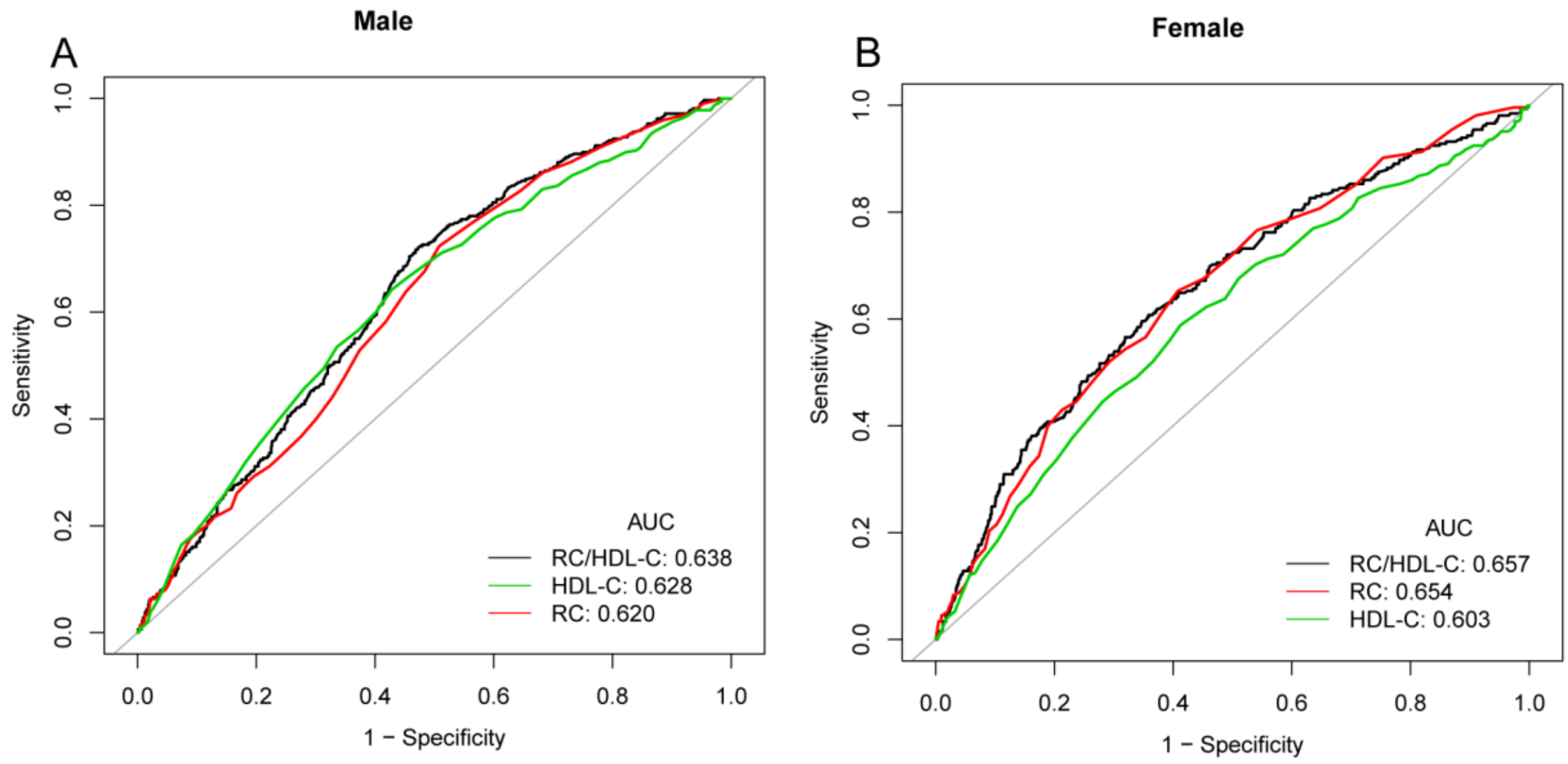

Supplementary Figure 2. ROC curves for RC/HDL-C ratio, compared to HDL-C and RC for HUA onset among males (A) and females (B). The predictive value for the RC/HDL-C ratio is more significant than other factors, as determined by its AUC.
